# Physical Activity Trajectories and Functional Recovery After Acute Stroke Among Adults in Sweden

**DOI:** 10.1001/jamanetworkopen.2023.10919

**Published:** 2023-05-01

**Authors:** Dongni Buvarp, Adam Viktorisson, Felix Axelsson, Elias Lehto, Linnea Lindgren, Erik Lundström, Katharina S. Sunnerhagen

**Affiliations:** 1 Rehabilitation Medicine Research Group, Department of Clinical Neuroscience, Institute of Neuroscience and Physiology, University of Gothenburg, Sweden; 2 Sahlgrenska University Hospital, Gothenburg, Sweden; 3 Department of Medical Sciences, Neurology, Uppsala University, Uppsala, Sweden

## Abstract

**Importance:**

The optimum level and timing of poststroke physical activity interventions to enhance functional recovery remain unclear.

**Objective:**

To assess the level of physical activity in the first 6 months after stroke among individuals with similar physical activity patterns over time and to investigate the association between physical activity trajectories and functional recovery at 6 months after stroke.

**Design, Setting, and Participants:**

This cohort study obtained data from the Efficacy of Fluoxetine–a Randomized Controlled Trial in Stroke, which was conducted in 35 stroke and rehabilitation centers across Sweden from October 2014 to June 2019. Adult participants (aged >18 years) were recruited between 2 and 15 days after stroke onset and followed up for 6 months. Participants who withdrew or were lost to follow-up were excluded from the longitudinal analysis. Data analyses were performed between August 15 and October 28, 2022.

**Exposures:**

Physical activity was assessed at 1 week, 1 month, 3 months, and 6 months. Multiple factors associated with physical activity trajectories were investigated. Association of the distinct trajectories with functional recovery was assessed in multivariable logistic regression.

**Main Outcomes and Measures:**

The primary outcomes were the distinct physical activity trajectories over time, which were identified using group-based trajectory modeling. The secondary outcome was the functional recovery at 6 months after stroke, which was assessed using the modified Rankin Scale.

**Results:**

Of the 1367 included participants (median [IQR] age, 72 years [65-79] years; 844 males [62%]), 2 distinct trajectory groups were identified: increaser (n = 720 [53%]) and decreaser (647 [47%]). The increaser group demonstrated a significant increase in physical activity level (mean difference, 0.27; linear slope β_1_ = 0.46; *P* < .001) and sustained it at light intensity from 1 week to 6 months, whereas the decreaser group showed a decline in physical activity and eventually became inactive (mean difference, −0.26; linear slope β_1_ = 1.81; *P* < .001). Male participants and those with normal cognition had higher odds of being in the increaser group, regardless of stroke severity. Increasing physical activity and sustaining it at light intensity were associated with a good functional outcome at 6 months (adjusted odds ratio, 2.54; 99% CI, 1.72-3.75; *P* < .001).

**Conclusions ad Relevance:**

Results of this study suggest that increased physical activity was associated with functional recovery 6 months after stroke. Interventions targeting individuals with decreasing physical activity in the subacute phase of stroke may play a role in improved functional outcomes.

## Key Points

**Question:**

In the first 6 months after a stroke, is there an association between physical activity trajectories and functional recovery?

**Findings:**

In this cohort study of 1367 participants who were stratified into 2 physical activity trajectory groups of increaser and decreaser, males and those with normal cognition had a higher likelihood of increasing and sustaining poststroke physical activity, regardless of stroke severity. Increased and sustained activity poststroke were associated with a good functional outcome at 6 months.

**Meaning:**

Findings of this study suggest that interventions targeting individuals with decreasing physical activity in the subacute phase of stroke may play a role in improved functional outcomes.

## Teaser

This cohort study examines whether increasing and decreasing physical activity or exercise is associated with functional recovery at 6 months after a stroke.

## Introduction

The health benefits of physical activity have been proven to considerably reduce the risk of all-cause mortality and cardiovascular disease, including stroke.^1,2,3^ Most stroke survivors experience long-term psychological and physical problems, and being physically active after stroke can promote better functional capacity and stroke recovery.^4,5^ Physical activity is associated with improved participation in and adjustment to life after stroke.^6^

Patients with stroke are susceptible to being less active than older adults with nonneurological chronic conditions, especially compared with healthy older individuals.^7,8^ Approximately 40% of stroke survivors reported being physically inactive 1 year after stroke.^9^ Understanding physical activity behavior over time is essential to maintaining a physically active life despite chronic conditions after stroke and retaining the benefits of rehabilitation interventions after discharge.

Longitudinal studies are crucial to determining the clinical factors associated with physical activity following stroke. This knowledge explains how early interventions to address physical activity participation should be targeted. A few small-sample longitudinal studies showed that patients with stroke were initially physically active during rehabilitation but tended to be inactive after discharge.^10,11^ However, most of these studies did not account for time factors and within-person variation over the long term. Thus, it is of clinical interest to explore the trajectories of physical activity over time to capture long-term heterogeneity and its association with functional recovery. This insight would enable the identification of a potential point of decline in physical activity and allow interventions that target the right persons at the right intensity and at the right time to enhance functional outcomes after stroke. In this study, we aimed to assess the level of physical activity in the first 6 months after stroke among individuals with similar physical activity patterns over time and to investigate the association between physical activity trajectories and functional recovery at 6 months after stroke.

## Methods

### Study Population and Design

Longitudinal data of the study participants were prospectively collected from the Efficacy of Fluoxetine—a Randomized Controlled Trial in Stroke (EFFECTS), a multicenter, placebo-controlled, double-blind randomized clinical trial that assessed the safety and efficacy of fluoxetine for functional recovery after stroke in 1500 randomized participants from 35 stroke centers and rehabilitation units across Sweden between October 2014 to June 2019.^12^ The sample size estimation was calculated and reported elsewhere.^12^ No significant differences in modified Rankin Scale (mRS) scores between the randomized groups were reported.^12^ This cohort study was approved by the medical ethics committee in Stockholm and by the Swedish Medical Agency, and it was conducted in accordance with the Declaration of Helsinki.^13^ All participants provided written informed consent. We followed the Strengthening the Reporting of Observational Studies in Epidemiology (STROBE) reporting guideline.^14^

Individuals were eligible to participate in EFFECTS if they were older than 18 years and had a clinical diagnosis of ischemic or hemorrhagic stroke within the past 2 to 15 days and at least 1 persistent neurological deficit.^12^ In the present study, 3 participants with an incorrect stroke diagnosis were excluded. Details of the inclusion process are available in the published protocol and its amendments.^12,15,16^

### Clinical Measurements

Baseline characteristics included age, sex, socioeconomic factors (living arrangements, educational level, and employment status), stroke type, stroke-related comorbidities (coronary artery disease and diabetes) and use of antihypertensive, antihyperlipidemic, anticoagulant, and antiplatelet drugs, and the total number of drugs (eTable 1 in Supplement 1). Stroke severity at randomization was assessed using the National Institutes of Health Stroke Scale (NIHSS)^17^ and was categorized into 3 sublevels: very mild (NIHSS score 0-3),^18^ mild (NIHSS score 4-5),^19^ or moderate to severe (NIHSS score ≥6). Whether participants had aphasia, were able to lift both arms, and were able to walk unaided at the time of randomization were recorded. Cognitive function was measured using the Montreal Cognitive Assessment (MoCA), with a maximum score of 30 points and the highest scores indicating normal cognition.^20^ Each individual MoCA score was then compared with the normative MoCA data derived from a large Swedish population after adjusting for age, sex, and educational level.^21^ Cognitive impairment was defined as a total MoCA score that was 1 SD or more below the normative mean, as suggested by a previous study,^21^ indicating that mild neurocognitive disorders were typically observed within the 1 to 2 SD range.

Physical activity was assessed at 1 week, 1 month, and 3 months after stroke through face-to-face or telephone interviews and at 6 months via physiotherapist follow-up. Physical activity was rated using the Saltin-Grimby Physical Activity Level Scale (SGPALS),^22,23^ which consists of 4 levels: inactivity (SGPALS 1); light-intensity physical activity for at least 4 hours per week (SGPALS 2); moderate-intensity physical activity, involving regular activity and training for at least 3 hours per week (SGPALS 3); and vigorous-intensity physical activity, involving regular hard training for competitive sports at least 4 hours per week (SGPALS 4). During the assessments, the participants were asked a series of standardized questions about their physical activity level since the most recent visit. For activities that were not explicitly mentioned by the SGPALS, the intensity level of the physical activity was rated using its respective metabolic equivalent of task value.^24^ Details of the assessment of physical activity level are provided in the eAppendix in Supplement 1.

Functional recovery was measured using the 7 ordinal-level mRS score, ranging from 0 to 6, with 0 indicating no disability, 5 indicating severe disability, and 6 indicating death.^25^ The association of physical activity trajectories with functional recovery was assessed using multivariable logistic regression.

### Statistical Analysis

#### Physical Activity Trajectories

Participants were excluded from the analysis if they were missing data on more than 30% of the outcome measures or if they had 2 or more missed follow-ups. The primary outcomes were the distinct physical activity trajectories over time. Physical activity trajectories were identified using group-based trajectory modeling, a data-derived statistical approach that does not rely on predefined subgroups.^26^ This method enables the classification of individuals into distinct subgroups based on similar physical activity patterns over time, thereby optimizing the focus on the outcome associated with specific subpopulations. The optimal number of trajectory groups and the degree of polynomials in each trajectory group were selected based on the lowest bayesian information criterion.^27^ All trajectory models consisting of 2-trajectory groups up to 6-trajectory groups were fitted until the best model was obtained (eMethods; eTables 2 and 3 in Supplement 1).^27^

Descriptive statistics were presented as medians with IQRs, number of participants with percentages, or means with SDs, as appropriate. To compare the distribution of clinical characteristics by trajectory group, we used the Mann-Whitney test for nonparametric variables; Pearson χ^2^, Fisher exact test, or Cochran Q for nominal variables; and Jonckheere-Terpstra test for ordinal variables, as appropriate.

#### Functional Outcome

The secondary outcome was the functional outcome 6 months after stroke, which was assessed using mRS scores. The scores were dichotomized into 2 groups: good outcome (0-2) vs poor outcome (3-6). Logistic regression models were applied to assess the association between trajectory groups and functional recovery while adjusting for covariates. Multicollinearity was assessed between all covariates using the variance inflation factor, and any covariates with a value exceeding 5 were excluded. All covariates were selected based on clinical reasoning and were included in the models without any predetermined variable selection. Subgroup analyses were also conducted by exploring the interaction effects between each specific subgroup and trajectory group in the logistic regression models. Odds ratios (ORs) for subgroup-specific associations and 99% CIs were reported.

All statistical tests were 2-sided, and the significance threshold was *P* < .01 to avoid a type 1 error due to multiple comparisons. Statistical analyses were performed between August 15 and October 28, 2022, using SAS, version 9.4 and the Proc Traj plug-in (SAS Institute Inc), for group-based trajectory modeling.^26^

We performed a sensitivity analysis by calculating the E-value for unmeasured confounding. The E-value indicates the minimum strength of the association an unmeasured confounder would need to have with both the outcome and the exposure to fully explain the observed association.^28^

## Results

Of the 1497 included participants with stroke at baseline, 46 died within 6 months. After excluding those who withdrew (n = 11) or were lost to follow-up (n = 119), the longitudinal study sample comprised 1367 participants with 5146 assessments (eFigure 1 in Supplement 1). The participants had a median (IQR) age of 72 (65-79) years and included 523 females (38%) and 844 males (62%) (Table 1). The excluded individuals were older (median [IQR] age, 77 [69-82] years; *P* < .001*)*; however, there were no significant differences in sex, stroke type, or stroke severity.

**Table 1.** Baseline Demographic Characteristics of the Study Population and by the Trajectory Group

Characteristic	No. (%)				
	Baseline study population (n = 1497)^a^	All participants (n = 1367)	Longitudinal sample (n = 1367)		
			Increaser group (n = 720)^b^	Decreaser group (n = 647)^c^	*P* value^d^
Age, median (IQR), y	73 (65-79)	72 (65-79)	70 (62-76)	74 (68-80)	<.001
Sex					
Male	922 (62)	844 (62)	492 (68)	352 (54)	<.001
Female	575 (39)	523 (38)	228 (32)	295 (46)	
Stroke type					
Ischemic	1312 (88)	1198 (87)	621 (86)	577 (89)	.10
Hemorrhagic	185 (12)	169 (12)	99 (14)	70 (11)	
OCSP classification^e^					
TACS	364 (24)	334 (24)	166 (27)	168 (29)	.34
PACS	594 (40)	542 (40)	283 (46)	259 (45)	
LACS	203 (14)	184 (14)	98 (16)	86 (15)	
POCS	126 (8)	114 (8)	60 (10)	54 (9)	
Unclear	25 (2)	24 (2)	14 (2)	10 (2)	
Living arrangements^f^					
Living alone	548 (37)	491 (36)	238 (33)	253 (39)	.02
Living with someone	949 (63)	846 (64)	482 (67)	394 (61)	
Employment status					<.001
Full-time	312 (21)	300 (22)	208 (29)	92 (14)	
Retired	1079 (72)	968 (71)	453 (63)	515 (80)	
Others^g^	106 (7)	99 (7)	59 (8)	40 (6)	
Educational level^h^					
≤12 y	769 (51)	695 (51)	395 (55)	300 (46)	.002
>12 y	728 (49)	672 (49)	325 (45)	347 (54)	
NIHSS score, median (IQR)	3 (2-6)	3 (2-6)	3 (2-6)	3 (2-6)	.35
0-3: Very mild stroke	536 (36)	491 (36)	262 (36)	229 (35)	
4-5: Mild stroke	525 (35)	472 (35)	256 (36)	216 (33)	
≥6: Moderate to severe stroke	436 (29)	404 (29)	202 (28)	202 (31)	
Cognition^i^					
Normal	615 (41)	571 (42)	355 (49)	216 (33)	<.001
Impaired	635 (42)	588 (43)	270 (38)	318 (49)	
Unclear	247 (17)	208 (15)	95 (13)	113 (18)	
Aphasia	267 (18)	239 (18)	119 (17)	120 (19)	.33
Able to lift both arms off beds	1171 (78)	1069 (78)	563 (78)	506 (78)	.99
Able to walk unaided	787 (53)	723 (53)	405 (56)	318 (49)	.009
Total No. of drugs used					
0-5	757 (51)	700 (51)	409 (57)	291 (45)	<.001
>5	740 (49)	667 (49)	311 (43)	356 (55)	
Diabetes comorbidity	299 (20)	272 (20)	128 (18)	144 (22)	.04
CAD comorbidity	234 (16)	212 (16)	96 (13)	116 (18)	.02
Antihypertensive drugs	1256 (84)	1148 (84)	583 (81)	565 (87)	.001
Antihyperlipidemic drugs	1204 (80)	1105 (81)	572 (79)	533 (82)	.17
Anticoagulant drugs	439 (29)	385 (28)	174 (24)	211 (33)	<.001
Antiplatelet drugs	991 (66)	921 (67)	507 (70)	414 (64)	.02
mRS score at 6 mo, median (IQR)^j^	1 (1-3)	1 (1-3)	1 (0-2)	2 (1-3)	<.001
0-2: Good outcome	941 (63)	890 (65)	544 (76)	346 (54)	
3-6: Poor outcome	536 (36)	469 (34)	173 (24)	296 (46)	
SGPALS rating after stroke, median (IQR)^k^					
1 wk	1 (1-2)	1 (1-2)	1 (1-2)	1 (1-2)	<.001
1 mo	1 (1-2)	1 (1-2)	2 (1-2)	1 (1-2)	<.001
3 mo	1 (1-2)	1 (1-2)	2 (1-2)	1 (1-1)	<.001
6 mo	1 (1-2)	1 (1-2)	2 (1-2)	1 (1-1)	<.001

Abbreviations: CAD, coronary artery disease; LACS, lacunar stroke; mRS, modified Rankin Scale; NIHSS, National Institutes of Health Stroke Scale; OCSP, Oxfordshire Community Stroke Project; PACS, partial anterior circulation stroke; POCS, posterior circulation stroke; SGPALS, Saltin-Grimby Physical Activity Level Scale; TACS, total anterior circulation stroke.

^a^ Sixty-four participants (4.2%) had a recurrent stroke during the first 6 months. Other conditions after the index hospitalization were previously reported elsewhere.^12^ Of the 1497 participants, 79 (5.2%) stayed in inpatient rehabilitation units.

^b^ The increaser group had a significant increase in physical activity from 1 week to 6 months after randomization and sustained it at light intensity.

^c^ The decreaser group had a significant decline in physical activity from 1 week to 6 months after randomization and became inactive.

^d^ Comparison between the increaser and decreaser groups was conducted using Jonckheere-Terpstra test, Pearson χ^2^, or Mann-Whitney test, as appropriate.

^e^ Not applicable in participants with hemorrhagic stroke (n = 185).

^f^ One participant had an unclear living arrangement.

^g^ Others included part-time (n = 68), unemployed (n = 15), volunteer (n = 2), or unknown (n = 21).

^h^ There were 197 participants with missing educational level data.

^i^ Cognition was assessed using Montreal Cognitive Assessment, which could not be conducted in 247 participants due to aphasia (n = 26); impairments, including diplopia, hemiparesis, fatigue, or worse general condition (n = 140); unknown reason (n = 60); and administrative or logistics problems (n = 21). Impaired cognition was defined as a score of 1 SD or more below a normative mean after adjusting for age, sex, and educational level.

^j^ The mRS data were missing in 20 participants, including 5 who withdrew from the study and 15 who were lost to follow-up.

^k^ The SGPALS data were missing in 167 participants at 1 week, 167 participants at 1 month, 104 participants at 3 months, and 124 participants at 6 months.

**Figure 1.**
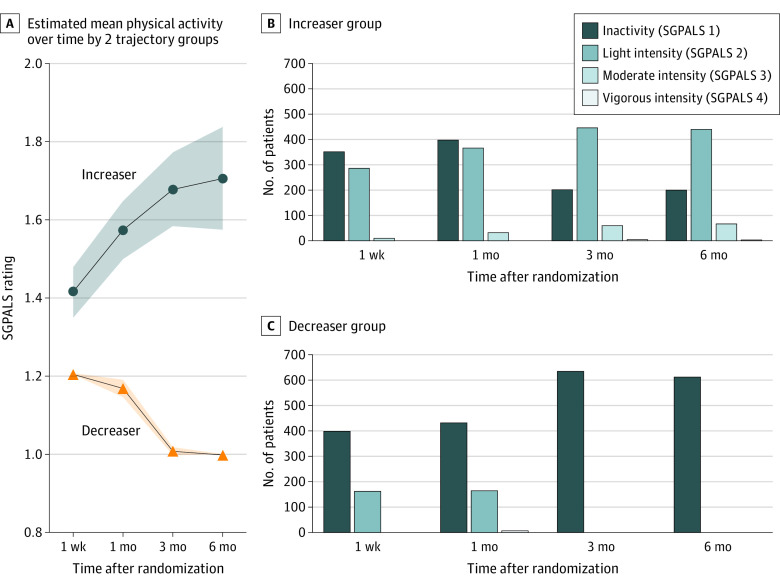
Increaser and Decreaser Groups Stratified by Physical Activity Patterns Over Time A, The increaser group had a significant increase in physical activity and sustained it at light intensity. The decreaser group had a significant decline in physical activity and became inactive. Shading represents CIs. B, The increaser group had a significant decrease in inactivity from 1 week to 6 months after randomization. C, The decreaser group had a significant increase in inactivity from 1 week to 6 months after randomization. SGPALS indicates Saltin-Grimby Physical Activity Level Scale.

Two distinct trajectory groups were identified: increaser (720 of 1367 participants [53%]) and decreaser (647 [47%]). The increaser group was characterized by a significant increase in physical activity (mean difference, 0.27; linear slope β_1_ = 0.46; *P* < .001) (Figure 1). The increaser group sustained light-intensity physical activity after achieving a maximum rate of increase between 1 week and 1 month (mean difference, 0.15; *P* < .001). The decreaser group was characterized by a significant decline in physical activity compared with the increaser group (intercept β_0_ = −0.63 vs 0.64; *P* < .001) (Figure 1) and eventually became inactive within 6 months (mean difference, −0.26; linear slope β_1_ = 1.81; *P* < .001). In general, both groups had a decelerating rate of change in physical activity over time (quadratic slope β_2_ = −0.06, *P* = .003 for increaser group; β_2_ = −0.65, *P* < .001 for decreaser group).

The participants in the increaser group were younger, were predominately male, worked full-time, had an educational level of 12 years or less, had normal cognition, were able to walk unaided, had no use of any antihypertensive or anticoagulant drugs, and used a total of fewer than 5 drugs compared with participants in the decreaser group (Table 1). Male participants had an adjusted 57% higher odds of being in the increaser group (adjusted OR [aOR], 1.57; 99% CI, 1.14-2.15; *P* < .001) (Table 2). Participants with cognitive impairment or unclear cognitive status had lower odds of being in the increaser group (aOR, 0.65 [99% CI, 0.46-0.91, *P* = .001] for impaired cognition; aOR, 0.59 [99% CI, 0.37-0.95, *P* = .004] for unclear cognitive status) (Table 2). There was no significant difference in stroke severity between the increaser and decreaser groups.

**Table 2.** Multivariable Logistic Regression of Covariates Associated With Good Functional Outcome at 6 Months After Stroke

Variable	Factors for increaser group^a^		Factors for good outcome			
	aOR (99% CI)^b^	*P* value	Crude OR (99% CI)	*P* value	aOR (99% CI)^c^	*P* value
Sex						
Female	1 [Reference]	<.001	1 [Reference]	<.001	1 [Reference]	.91
Male	1.57 (1.14-2.15)		1.51 (1.12-2.04)		1.02 (0.68-1.52)	
Age group, y						
≤70	1 [Reference]	NA	1 [Reference]	NA	1 [Reference]	NA
71-74	0.92 (0.56-1.50)	.66	0.63 (0.40-0.99)	.009	0.95 (0.50-1.77)	.82
75-80	0.78 (0.49-1.23)	.16	0.41 (0.28-0.61)	<.001	0.57 (0.32-1.01)	.02
>80	0.63 (0.38-1.05)	.02	0.25 (0.17-0.38)	<.001	0.43 (0.23-0.80)	<.001
Stroke type						
Hemorrhagic	1 [Reference]	.17	1 [Reference]	.02	1 [Reference]	.61
Ischemic	0.70 (0.36-1.37)		1.46 (0.95-2.25)		0.85 (0.37-1.94)	
Living arrangements						
Living alone	0.98 (0.71-1.35)	.86	0.98 (0.72-1.33)	.85	1.49 (0.99-2.25)	.02
Living with someone	1 [Reference]		1 [Reference]		1 [Reference]	
Employment status						
Full time	1.37 (0.73-2.59)	.20	1.36 (0.65-2.84)	.28	1.30 (0.53-3.17)	.45
Retired	0.87 (0.47-1.63)	.57	0.42 (0.22-0.81)	<.001	0.54 (0.23-1.27)	.06
Others^d^	1 [Reference]	NA	1 [Reference]	NA	1 [Reference]	NA
Educational level						
≤12 y	1 [Reference]	.10	1 [Reference]	.33	1 [Reference]	.08
>12 y	0.82 (0.59-1.12)		0.90 (0.67-1.20)		1.32 (0.87-1.98)	
NIHSS score						
0-3: Very mild stroke	0.90 (0.53-1.53)	.59	8.30 (5.52-12.48)	<.001	2.22 (1.18-4.19)	.001
4-5: Mild stroke	1.02 (0.64-1.64)	.90	4.41 (3.03-6.42)	<.001	1.62 (0.95-2.77)	.02
≥6: Moderate to severe stroke	1 [Reference]	NA	1 [Reference]	NA	1 [Reference]	NA
Aphasia	0.99 (0.66-1.50)	.97	0.89 (0.60-1.30)	.41	1.10 (0.66-1.82)	.64
Cognition						
Normal	1 [Reference]	NA	1 [Reference]	NA	1 [Reference]	NA
Impaired	0.65 (0.46-0.91)	.001	0.27 (0.19-0.38)	<.001	0.37 (0.24-0.58)	<.001
Unclear	0.59 (0.37-0.95)	.004	0.27 (0.17-0.43)	<.001	0.65 (0.36-1.16)	.06
Able to lift both arms	0.85 (0.52-1.39)	.39	6.41 (4.43-9.27)	<.001	2.91 (1.64-5.17)	<.001
Able to walk unaided	1.20 (0.83-1.74)	.21	7.18 (5.13-10.06)	<.001	3.53 (2.26-5.51)	<.001
Total No. of drugs used						
≤5	1 [Reference]	.23	1 [Reference]		1 [Reference]	
>5	0.86 (0.61-1.19)		0.48 (0.36-0.65)	<.001	0.67 (0.44-1.02)	.02
Diabetes comorbidity	0.83 (0.57-1.23)	.23	0.62 (0.43-0.89)	<.001	0.61 (0.38-0.99)	.009
CAD comorbidity	0.91 (0.59-1.39)	.55	0.70 (0.47-1.04)	.02	1.04 (0.61-1.75)	.86
Antihypertensive drugs	0.78 (0.51-1.19)	.13	0.72 (0.47-1.09)	.04	0.91 (0.52-1.60)	.67
Antihyperlipidemic drugs	0.87 (0.55-1.38)	.43	1.52 (1.06-2.19)	.003	1.30 (0.73-2.30)	.24
Anticoagulant drugs	0.87 (0.56-1.34)	.39	0.46 (0.33-0.63)	<.001	0.83 (0.49-1.39)	.34
Antiplatelet drugs	1.45 (0.90-2.36)	.05	1.68 (1.23-2.28)	<.001	1.17 (0.65-2.13)	.49
Trajectory of physical activity						
Increaser	NA	NA	3.23 (2.32-4.49)	<.001	2.54 (1.72-3.75)	<.001
Decreaser	NA	NA	1 [Reference]	NA	1 [Reference]	NA

Abbreviations: aOR, adjusted odds ratio; CAD, coronary artery disease; NA, not applicable; NIHSS, National Institutes of Health Stroke Scale; OR, odds ratio.

^a^ The increaser group had a significant increase in physical activity from 1 week to 6 months after randomization and sustained it at light intensity.

^b^ Model fit statistics included Hosmer-Lemeshow goodness of fit of 0.31; Akaike information criterion of 1893.16; Schwarz criterion of 1898.39; −2Log L of 1891.16; *R*^2^ of 0.09; and area under the curve of 0.67.

^c^ Model fit statistics included Hosmer-Lemeshow goodness of fit of 0.27; Akaike information criterion of 1250.93; Schwarz criterion of 1381.30; −2Log L of 1200.93; *R*^2^ of 0.33; and area under the curve of 0.85.

^d^ Others included part-time (n = 68), unemployed (n = 15), volunteer (n = 2), or unknown (n = 21).

### Association of Increased Physical Activity and Sustained Light-Intensity Activity With Good Outcomes at 6 Months

**Figure 2.**
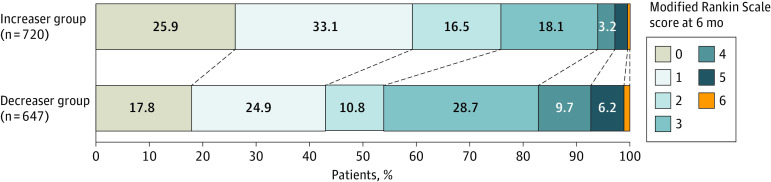
Distribution of the Increaser and Decreaser Groups by the Modified Rankin Scale Score There were significant differences between the increaser and decreaser groups with modified Rankin Scale scores of 0, 1, 2, 3, 4 and 5, but not 6.

Eight participants were excluded due to missing mRS data at 6 months (1 withdrew; 7 were lost to follow-up). Of the 1359 participants, 890 (65%) had a good outcome and 469 (35%) had a poor outcome at the 6-month assessment. Among the participants with a good outcome, 544 (61%) were in the increaser group and 346 (39%) were in the decreaser group. There were significant differences between the increaser and decreaser group in the degree of disability (mRS scores 0 to 5), except for mRS score 6 (Figure 2).

**Figure 3.**
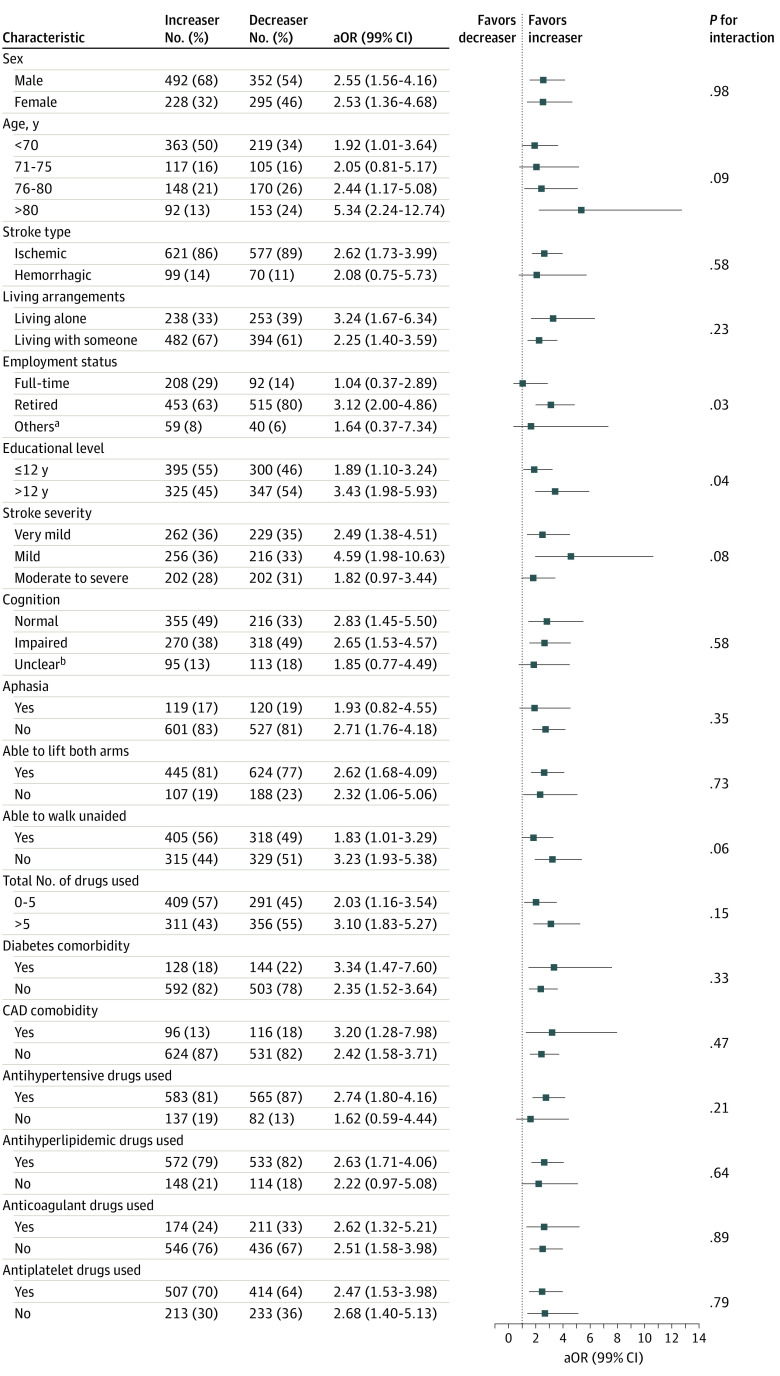
Interaction Analysis Between Characteristics and the Trajectory Groups Adjusted odds ratios (aORs) indicated that the increaser group was more likely to have a good outcome (modified Rankin Scale score 0-2). Significance level was *P* < .01. CAD indicates coronary artery disease. ^a^Others included part-time (n = 63), unemployed (n = 14), or volunteer (n = 2). ^b^Montreal Cognitive Assessment could not be conducted in 208 participants due to aphasia (n = 19); impairments, including diplopia, hemiparesis, fatigue, or worse general condition (n = 126); unknown reason (n = 50); and administrative or logistics problems (n = 13).

Covariates associated with functional outcomes at 6 months are shown in Table 2. The increaser group had higher adjusted odds of a good outcome than the decreaser group (aOR, 2.54; 99% CI, 1.72-3.75; *P* < .001). Age older than 80 years (aOR, 0.43; 99% CI, 0.23-0.80; *P* < .001), having diabetes (aOR, 0.61; 99% CI, 0.38-0.99; *P* = .009), and having impaired cognition (aOR, 0.37; 99% CI, 0.24-0.58; *P* < .001) were associated with lower adjusted odds of a good outcome. Experiencing very mild stroke (aOR, 2.22; 99% CI, 1.18-4.19; *P* = .001), being able to lift both arms (aOR, 2.91; 99% CI, 1.64-5.17; *P* < .001), and being able to walk unaided at randomization (aOR, 3.53; 99% CI, 2.26-5.51; *P* < .001) were associated with higher adjusted odds of a good outcome. Although a good outcome was shown to be more favorable among the increaser group in all covariate subgroups, the results of the interaction effects analysis were not statistically significant (Figure 3). In the sensitivity analysis, the E-value for an OR of 2.54 was 2.57 and the lower confidence bound was 1.95. This finding suggested that the magnitude of unmeasured confounder would need to be large to explain the observed associations between physical activity trajectory and functional outcome (eFigure 2 in Supplement 1).

## Discussion

In this longitudinal and prospective cohort study, 2 distinct physical activity trajectories were identified. The increaser group increased their physical activity and sustained it at light intensity, while the decreaser group decreased their physical activity and became inactive. Male participants and those with normal cognition had a higher likelihood of increasing physical activity, regardless of stroke severity. We found an association between increased physical activity and a good functional outcome at 6 months after stroke.

Overall, the study population had low physical activity levels, with approximately half of the participants being inactive. Among those who increased their physical activity, only a small number (Figure 1) engaged in moderate- to vigorous-intensity exercise, which was a lower percentage than what has been reported in previous studies (approximately 40% active),^9,29,30^ despite most participants experiencing a mild stroke. The decreaser group had a maximum decrease rate between 1 and 3 months and was followed by a plateau, which was consistent with results from previous studies.^10,11^ This finding suggested a potential decline in motivation after discharge despite the importance of promoting physical activity after stroke as outlined in several clinical guidelines.^31^ Establishing physical activity behavior early may enhance the translation of functional gains achieved during rehabilitation into long-term benefits.^32^ Hence, it may be advisable for clinicians to target the potential decreaser (ie, female participants with cognitive impairment at discharge) to emphasize the functional benefits of maintaining physical activity, which may facilitate the translation of knowledge into action, before the plateau occurs. Another explanation could be that people who were physically active prior to stroke might be more likely to continue being active after the stroke, although previous research has reported mixed results.^29,33^

We found that male participants had a higher likelihood of increasing physical activity over time, which was in line with previous research.^34,35,36^ Nevertheless, the existence of a sex-based gap in physical activity is widely acknowledged and may be partially associated with differences in individual perceptions of physical activity power, which may vary even within sexes.^37^ The barriers to females’ involvement in physical activity are numerous, including environmental, psychological, and sociocultural factors, which result in less likelihood to engage in activities that are vigorous, require skill, and are done outdoors.^38^ However, some studies have found no significant sex-based differences in physical activity after stroke.^8,39^ This finding suggests that females may have had behavioral changes to some extent after stroke. Recognizing the sex-based differences in physical activity after a stroke is crucial in mitigating potential barriers for females and motivating behavioral changes for inactive individuals.

The study findings suggest that preserved cognition after stroke is necessary to sustain or achieve a higher level of physical activity. This finding is also supported by previous studies.^3,40^ It is expected that processing and performing activities of vigorous intensity require higher demands on cognitive and executive functions.^41^ Early interventions aimed at promoting physical activity may be necessary to mitigate cognitive decline given that prior research has indicated that engaging in light- to moderate-intensity physical activity can be beneficial for cognitive improvement.^40^ Moreover, the present study demonstrated that increasing physical activity and normal cognition are both significantly and independently associated with better functional outcome. Thus, special interventions may be required in improving outcomes for stroke survivors with cognitive impairment.

Unexpectedly, we found that stroke severity was not a significant factor of increased physical activity. This finding is encouraging, suggesting that patients with stroke can gain functional benefits by becoming more physically active regardless of stroke severity. Good functional recovery was also observed in the increaser group at different levels of stroke severity, despite the lack of significance. This finding may indicate that stroke severity and physical activity have isolated associations with functional recovery that are greater in magnitude than the sum of both associations. While it may be expected for patients with severe stroke to have poorer functional recovery despite their physical activity level, being physically active is still associated with a better outcome, regardless of stroke severity, supporting the health benefits of poststroke physical activity.

The findings from this study indicate that increased physical activity of at least light intensity during the subacute phase after stroke was associated with a good functional outcome at 6 months, which is consistent with earlier findings in patients with mild or moderate stroke.^5,6^ However, no significant interactions were found in the subgroup analysis, indicating that no other covariate traits could modify the association between the physical activity trajectories and functional outcome at 6 months after stroke. Early rehabilitation is beneficial as the optimal window for increasing recovery becomes narrower with time.^42^ Increased physical activity has been shown at the behavioral and molecular levels to be associated with enhanced neuroprotectivity and neuroplasticity.^43^ Light-intensity physical activity might already play an optimal role at the early subacute phase in improved functional recovery before spontaneous recovery diminishes.

### Limitations

This study has several limitations. First, there was a high proportion of participants with mild stroke, which may limit the generalizability of the findings to populations with mild-to-moderate stroke severity. This situation may also limit the ability to replicate the study findings. Second, although group-based trajectory is an emerging statistical method in research, the analysis was able to identify only 2 trajectory groups (increaser and decreaser) in this study, which may indicate that the patient group was rather homogenous. Furthermore, we may have lost information on patients with fluctuating physical activity levels since they were stratified in either the increaser or decreaser group. Third, multiple factors with several dimensions were included; however, due to limited sample size, there may be confounding factors that were not included in this study, such as body mass index, diet, prestroke physical activity level, and recurrent stroke. Fourth, although we documented participants’ physical training with a physiotherapist, an occupational therapist, or on their own, almost all individuals had a combination of training types. Consequently, it was not possible to separate the outcomes from the different types of training in any meaningful way to fully evaluate their impact. Fifth, SGPALS was rated through interviews using standardized questions, which might be prone to recall and emotional bias.

## Conclusions

This large longitudinal, prospective cohort study found that increased physical activity sustained at light intensity for more than 4 hours per week during the subacute phase of stroke was associated with a good outcome at 6 months. Males and patients with normal cognition had a higher likelihood of increasing their physical activity after stroke, regardless of stroke severity at admission. The study findings suggest that interventions targeting individuals with decreasing physical activity in the subacute phase of stroke may play a role in functional recovery.

## Notes

Supplement 1. **eAppendix.** The Assessment of Physical Activity Using Saltin-Grimby Physical Activity **eTable 1.** Anatomical Therapeutic Chemical (ATC) Classification Codes **eMethods.** Group-Based Trajectory Model Building Selection and Model Evaluation **eTable 2.** The 2-Trajectory Group With Different Polynomial Orders **eTable 3.** Diagnostic Criteria for the Final Model **eFigure 1**. Flow Chart of Participants in the Longitudinal Study **eFigure 2.** Estimated E-Value for an Odds Ratio of 2.54 and Outcome Prevalence >15%
eReferences

Supplement 2.
Data Sharing Statement
