# Supplementary material for: Physical Activity Trajectories and Functional Recovery After Acute Stroke Among Adults in Sweden
Source: JAMA Netw Open. 2023 May 1;6(5):e2310919. doi: 10.1001/jamanetworkopen.2023.10919 (PMC10152305; doi:10.1001/jamanetworkopen.2023.10919)
Supplement: Supplement 1. — eAppendix. The Assessment of Physical Activity Using Saltin-Grimby Physical Activity eTable 1. Anatomical Therapeutic Chemical (ATC) Classification Codes eMethods. Group-Based Trajectory Model Building Selection and Model Evaluation eTable 2. The 2-Trajectory Group With Different Polynomial Orders eTable 3. Diagnostic Criteria for the Final Model eFigure 1. Flow Chart of Participants in the Longitudinal Study eFigure 2. Estimated E-Value for an Odds Ratio of 2.54 and Outcome Prevalence >15% eReferences [file jamanetwopen-e2310919-s001.pdf]

Supplemental Online Content

Buvarp D, Viktorisson A, Axelsson F, et al. Physical activity trajectories and functional recovery after acute stroke among adults in Sweden. *JAMA Netw Open*. 2023;6(5):e2310919. doi:10.1001/jamanetworkopen.2023.10919

eAppendix. The Assessment of Physical Activity Using Saltin-Grimby Physical Activity

eTable 1. Anatomical Therapeutic Chemical (ATC) Classification Codes

eMethods. Group-Based Trajectory Model Building Selection and Model Evaluation

eTable 2. The 2-Trajectory Group With Different Polynomial Orders

eTable 3. Diagnostic Criteria for the Final Model

eFigure 1. Flow Chart of Participants in the Longitudinal Study

eFigure 2. Estimated E-Value for an Odds Ratio of 2.54 and Outcome Prevalence > 15%

eReferences

This supplemental material has been provided by the authors to give readers additional information about their work.

© 2023 Buvarp D et al. *JAMA Network Open.*

eAppendix. The assessment of physical activity using Saltin-Grimby Physical activity.

The use of the original Saltin-Grimby Physical Activity Scale (SGPALS)

The duration and intensity of the exercise with the physiotherapist, exercise with other occupational categories, and exercise at home were separately documented. The collected data on physical activity were graded according to the different levels of physical activity presented in the updated version of SGPALS.[1,2] The criteria for SGPALS 3 and 4 were specified by determining a time requirement: at least 3 hours of moderate physical activity for SGPALS 3 and at least four hours of vigorous physical activity for SGPALS 4.

If the reported activities were not explicitly mentioned in the SGPALS, the metabolic equivalent of task (MET) values from the 2011 Compendium of Physical activities[3] were used to determine the intensity of the activity. The intensity required for SGPALS 3 was set to a MET value of 3-6 and for SGPALS 4 to a MET value > 6.

If several activities were reported during the same period, the least intensive activity determined the intensity level and subsequently the SGPALS level. If the participant met the criteria for several levels of SGPALS, the highest intensity level determined the participant's level of physical activity.

© 2023 Buvarp D et al. *JAMA Network Open.*

eTable 1. Anatomical Therapeutic Chemical (ATC) classification codes

Medications	ATC codes
Antihypertensive drugs	Antihypertensives (C02), Diuretics (C03, Beta blocking agents (C07), Calcium channel blockers (C08), Agents acting on the renin-angiotensin system (C09)
Antihyperlipidemic drugs	HMG CoA reductase inhibitors (C10AA)
Anticoagulant drugs	Warfarin (B01AA03), Heparin group (B01AB), direct factor Xa inhibitors (B01AF) and direct thrombin inhibitors (B01AE)
Antiplatelet	Platelet aggregation inhibitors (B01AC)

© 2023 Buvarp D et al. *JAMA Network Open.*

eMethods. Group-based trajectory modeling building selection and model evaluation

The best-fit model was investigated stepwise. First, a single cubic trajectory model was tested. If the cubic function was not significantly different, a lower polynomial function was tested until statistical significance was obtained. The number of trajectories with polynomial order was determined using the Bayesian Information Criteria (BIC) and estimated proportions for the trajectory groups. The number of trajectories was tested from 2 up to 6 trajectory groups with cubic components. The 2-trajectory group with polynomial orders was statistically significant, and no group proportions were found under the 5% threshold.

Three diagnostic criteria were used to evaluate the adequacy of the selected models and the number of trajectory groups to ensure that each participant was accurately assigned[4]: 1) The average of the posterior probabilities of group membership (AvePP). This was calculated by averaging each individual's posterior probabilities of group membership assigned group membership based on the maximum-probability assignment rule. The threshold for the AvePP of each trajectory group exceeded 0.7, which is acceptable. 2) Odds of correct classification for each trajectory group, j, were calculated as follows: the odds of a correct classification exceeding a threshold of 5 are recommended. 3) The difference between the estimated posterior probabilities of group membership and the proportion of group membership was less than 50%. The results of the diagnostic criteria according to the trajectory groups are presented below.

$$OCC_j = \frac{AvePP_j}{1 - AvePP_j} / \frac{\pi_j}{1 - \pi_j}$$

© 2023 Buvarp D et al. *JAMA Network Open.*

eTable 2. The 2-trajectory group with different polynomial orders.

2-trajectory group with different polynomial order[a]	BIC (total assessments)	BIC (number of patients	AIC	Log(2ΔBIC)[b]
2 (1 0)	-5040.47	-5037.16	-5024.11	
2 (1 1)	-5028.45	-5024.47	-5008.81	26.1
2 (1 2)	-5024.88	-5020.24	-5001.97	4.23
2 (2 2)	-5004.49	-4999.18	-4978.30	21.06

[a] Only models with significant polynomial function are presented.

[b] Log(2ΔBIC) is the logged Bayes factor, calculated as 2 * ($BIC_{complex} - BIC_{previous}$). According to the suggested criteria for interpreting the estimate of the log(2ΔBIC), log(2ΔBIC) 0 to 2 indicates not worth mentioning, 2 to 6 indicates positive evidence for choosing the complex model, 6 to 10 represents strong evidence, and > 10 represents strong evidence.[4]

BIC, Bayesian Information Criteria; AIC, Akaike Information Criterion;

© 2023 Buvarp D et al. *JAMA Network Open.*

eTable 3. Diagnostic criteria for the final model

2-trajectory group with one quadratic and one quadratic	AvePP	OCCj	Difference between the estimated posterior probabilities and the proportion of group membership
Increaser	0.988	57.30	5.7
Decreaser	0.864	8.82	5.8

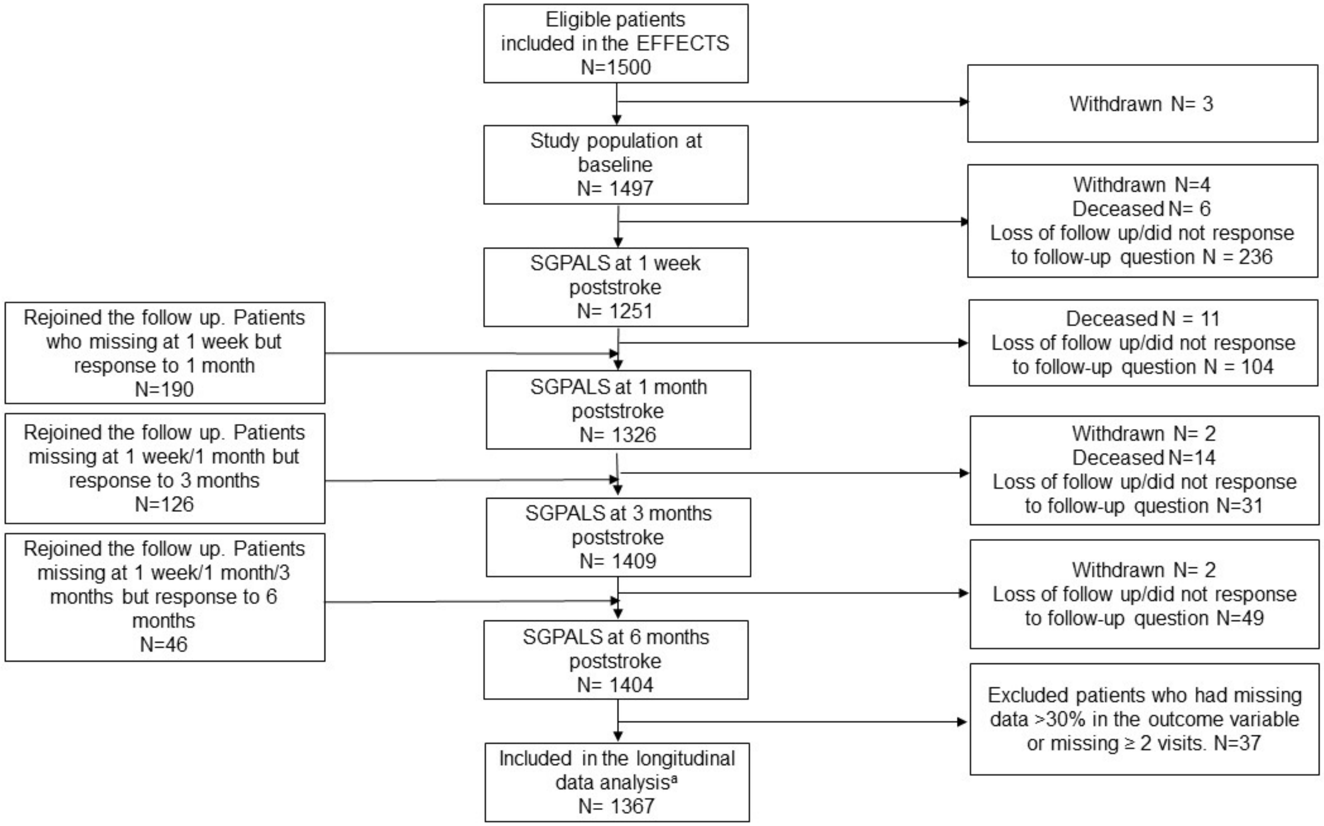

eFigure 1. Flow chart of participants in the longitudinal study.

[a] Of the 1367 patients in the longitudinal analysis, 10 participants who died after were also included.

© 2023 Buvarp D et al. *JAMA Network Open.*

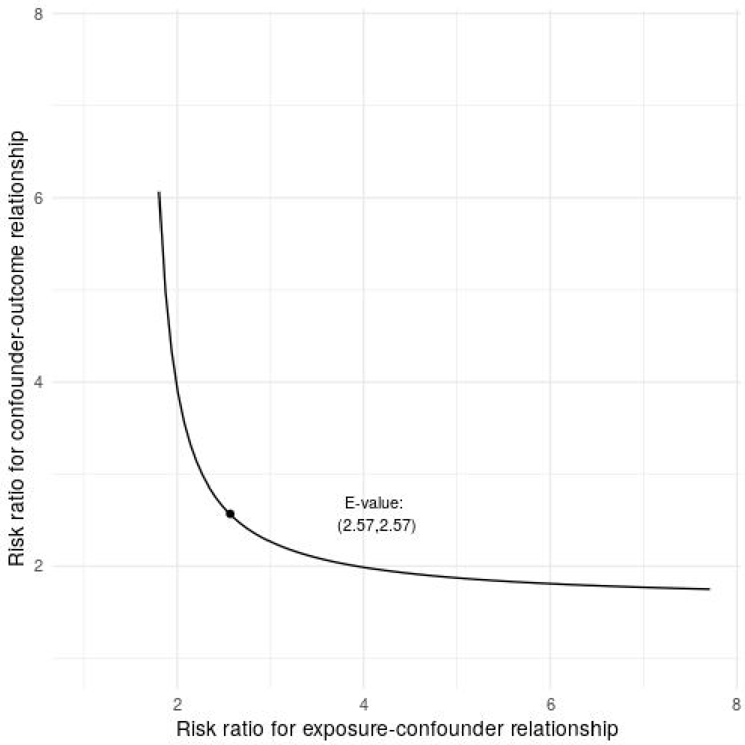

eFigure 2. Estimated E- value for an odds ratio of 2.54 and outcome prevalence > 15%.

The E-value was calculated by using the online calculator (https://www.hsph.harvard.edu/tylervanderweele/tools-and-tutorials/).[5]

© 2023 Buvarp D et al. *JAMA Network Open.*

eReferences

1. Saltin B, Grimby G. Physiological analysis of middle-aged and old former athletes: comparison with still active athletes of the same ages. *Circulation*. 1968;38(6):1104-11152.
2. Grimby G, Börjesson M, Jonsdottir I, Schnohr P, Thelle D, Saltin B. The "Saltin–Grimby physical activity level scale" and its application to health research. *Scand J Med Sci Sports*. 2015;25:119-125.3.
3. Ainsworth BE, Haskell WL, Herrmann SD, et al. 2011 Compendium of Physical Activities: A Second Update of Codes and MET Values. *Med Sci Sports Exerc*. 2011;43(8):1575-1581. doi:10.1249/MSS.0b013e31821ece12.
4. Nagin DS, Nagin D. *Group-based modeling of development*. Harvard University Press; 2005
5. VanderWeele TJ, Ding P. Sensitivity Analysis in Observational Research: Introducing the E-Value. *Ann. Intern.Med*. 2017/08/15 2017;167(4):268-274. doi:10.7326/M16-2607

© 2023 Buvarp D et al. *JAMA Network Open.*
