# Supplementary material for: Physical Activity Trajectories and Functional Recovery After Acute Stroke Among Adults in Sweden
Source: JAMA Netw Open. 2023 May 1;6(5):e2310919. doi: 10.1001/jamanetworkopen.2023.10919 (PMC10152305; doi:10.1001/jamanetworkopen.2023.10919)
Supplement: Supplement 2. — Data Sharing Statement [file jamanetwopen-e2310919-s002.pdf]

Data Sharing Statement

Buvarp. Physical Activity Trajectories and Functional Recovery After Acute Stroke Among Adults in Sweden. *JAMA Netw Open*. Published May 01, 2023.
doi:10.1001/jamanetworkopen.2023.10919

Data
Data available: No

Additional Information
Explanation for why data not available: Data may be available to researchers upon request, after a review of the secrecy. According to Swedish regulations (https://etikprovningsmyndigheten.se/), permission to use data can only be based on application and approval from the Swedish Ethics Review Authority.
